# The association between clinical laboratory data and chest CT findings explains disease severity in a large Italian cohort of COVID-19 patients

**DOI:** 10.1186/s12879-021-05855-9

**Published:** 2021-02-08

**Authors:** Simone Canovi, Giulia Besutti, Efrem Bonelli, Valentina Iotti, Marta Ottone, Laura Albertazzi, Alessandro Zerbini, Pierpaolo Pattacini, Paolo Giorgi Rossi, Rossana Colla, Tommaso Fasano, on behalf of the Reggio Emilia COVID-19 Working Group;, Massimo Costantini, Roberto Grilli, Massimiliano Marino, Giulio Formoso, Debora Formisano, Paolo Giorgi Rossi, Emanuela Bedeschi, Cinzia Perilli, Elisabetta La Rosa, Eufemia Bisaccia, Ivano Venturi, Massimo Vicentini, Cinzia Campari, Francesco Gioia, Serena Broccoli, Marta Ottone, Pierpaolo Pattacini, Giulia Besutti, Valentina Iotti, Lucia Spaggiari, Pamela Mancuso, Andrea Nitrosi, Marco Foracchia, Rossana Colla, Alessandro Zerbini, Marco Massari, Anna Maria Ferrari, Mirco Pinotti, Nicola Facciolongo, Ivana Lattuada, Laura Trabucco, Stefano De Pietri, Giorgio Francesco Danelli, Laura Albertazzi, Enrica Bellesia, Simone Canovi, Mattia Corradini, Tommaso Fasano, Elena Magnani, Annalisa Pilia, Alessandra Polese, Silvia Storchi Incerti, Piera Zaldini, Efrem Bonelli, Bonanno Orsola, Matteo Revelli, Carlo Salvarani, Carmine Pinto, Francesco Venturelli

**Affiliations:** 1 Clinical chemistry and Endocrinology Laboratory, Departement of Diagnostic Imaging and Laboratory Medicine, AUSL-IRCCS di Reggio Emilia, viale Risorgimento 80, 42123 Reggio Emilia, Italy; 2 Radiology Unit, Departement of Diagnostic Imaging and Laboratory Medicine, AUSL-IRCCS di Reggio Emilia, viale Risorgimento, 80, 42123 Reggio Emilia, Italy; 3 Clinical and Experimental Medicine PhD program, University of Modena and Reggio Emilia, Modena, Italy; 4 Epidemiology Unit, AUSL-IRCCS di Reggio Emilia, viale Risorgimento, 80, 42123 Reggio Emilia, Italy; 5 Laboratory of autoimmunity, allergology and innovative biotechnologies, Departement of Diagnostic Imaging and Laboratory Medicine, AUSL-IRCCS di Reggio Emilia, viale Risorgimento, 80, 42123 Reggio Emilia, Italy

**Keywords:** COVID-19, Clinical laboratory, Computed tomography

## Abstract

**Background:**

Laboratory data and computed tomography (CT) have been used during the COVID-19 pandemic, mainly to determine patient prognosis and guide clinical management. The aim of this study was to evaluate the association between CT findings and laboratory data in a cohort of COVID-19 patients.

**Methods:**

This was an observational cross-sectional study including consecutive patients presenting to the Reggio Emilia (Italy) province emergency rooms for suspected COVID-19 for one month during the outbreak peak, who underwent chest CT scan and laboratory testing at presentation and resulted positive for SARS-CoV-2.

**Results:**

Included were 866 patients. Total leukocytes, neutrophils, C-reactive protein (CRP), creatinine, AST, ALT and LDH increase with worsening parenchymal involvement; an increase in platelets was appreciable with the highest burden of lung involvement. A decrease in lymphocyte counts paralleled worsening parenchymal extension, along with reduced arterial oxygen partial pressure and saturation. After correcting for parenchymal extension, ground-glass opacities were associated with reduced platelets and increased procalcitonin, consolidation with increased CRP and reduced oxygen saturation.

**Conclusions:**

Pulmonary lesions induced by SARS-CoV-2 infection were associated with raised inflammatory response, impaired gas exchange and end-organ damage. These data suggest that lung lesions probably exert a central role in COVID-19 pathogenesis and clinical presentation.

**Supplementary Information:**

The online version contains supplementary material available at 10.1186/s12879-021-05855-9.

## Background

In December, 2019, an outbreak of a novel coronavirus (SARS-CoV-2) became apparent in China, and has since become a global concern for human health and a major challenge for national health services. In Italy, due to the overwhelming hospital influx of patients by the end of February 2020, there was serious concern regarding the national health system’s capacity to cope with severely infected subjects who required intensive care for the management of SARS-CoV-2-related pneumonia [1]. Clinical information, laboratory data and CT scans have been used in the setting of COVID-19 pandemic, peripherally as part of the diagnostic work-up, but mostly to help define patient prognosis and to guide clinical management.

A recent meta-analysis systematically compared laboratory findings in cohorts of patients grouped by disease severity or mortality [2]. Clear patterns of inflammatory, hematological, biochemical and immune biomarker abnormalities were found, warranting inclusion of laboratory parameters in risk stratification models.

CT scan provides information on both type and degree of parenchymal involvement, the first including mostly ground-glass opacities (GGO) and consolidation, and the latter by means of estimating, visually or automatically, the percentage of involved lung parenchyma. A potential prognostic role of chest CT findings, especially the extent of parenchymal involvement, has been proposed [3–6].

Few studies have also combined clinical, laboratory, and CT findings [7, 8]. Some of these suggest that the performance of the prognostic model is better when adding CT features, while the others show that CT findings had insufficient prognostic power to be used in combination models.

In this setting, it would be useful to understand how CT changes relate to laboratory data describing different clinical and pathophysiological derangements in COVID-19. Indeed, studies reporting the association between lung imaging and clinical laboratory data in COVID-19 patients are scarce and heterogeneous in terms of study population, laboratory tests and imaging interpretation. While consistent associations are mostly reported for some laboratory data [9–11] conflicting results have been described for others [5].

The aim of this study was to evaluate the association between laboratory data and chest CT findings in a large Italian cohort of COVID-19 patients.

## Methods

### Setting

The Reggio Emilia province (532,000 inhabitants), located in Northern Italy, counts six hospitals in its territory. The first case of SARS-CoV-2 infection occured on February 27, 2020, and up to June 14, 2020, there were 4950 virologically-confirmed cases. The study was approved by the Area Vasta Emilia Nord Ethics Committee on April 7, 2020, with protocol number 2020/0045199. Given the retrospective nature of the study, patients’ informed consent to participate in the study was obtained whenever possible.

### Study design and population

In this observational cross-sectional study, we included all consecutive patients who presented to the provincial emergency rooms (ERs) between February 27 and March 23 for suspected COVID-19, underwent chest CT scan and blood tests at ER presentation and tested positive for SARS-CoV-2 at RT-PCR within 10 days from ER presentation.

The diagnostic protocol for patients presenting to the ER for suspected COVID-19 in the province of Reggio Emilia has been previously described [12]. Briefly, it included RT-PCR, blood tests, chest X-rays, and chest CT scan in cases of suggestive X-ray or clinical findings.

### Data collection

Data were retrieved from the COVID-19 Surveillance Registry coordinated by the National Institute of Health and implemented in each Local Health Authority. This registry collects information about date of symptom onset, diagnosis, hospitalization and death or recovery of patients testing positive for SARS-CoV-2 RNA by RT-PCR. Information is directly collected from the patient himself/herself through daily telephone contact when cared for in an outpatient setting and from electronical medical records when hospitalized. Data from the COVID-19 Surveillance Registry were linked with the provincial Radiology Information System to search for CT scans performed at the moment of or after the onset of COVID symptoms. For all included patients, hospital discharge databases were linked to the COVID-19 Registry to identify hospital admissions in the 10 years preceding COVID-19 hospitalization in order to calculate the Charlson index for each patient [13].

### Blood tests and RT-PCR

Laboratory results for C-reactive protein (CRP), lactate dehydrogenase (LDH), white blood cells, lymphocytes, neutrophils, and platelets were measured on ER admission in the entire cohort. Results of arterial blood gas analysis were also measured. For patients presenting to ER up to March 13, results for total bilirubin, creatinine, aspartate transaminase (AST), alanine transaminase (ALT), albumin, procalcitonin and prothrombin time (PT) were also collected. Complete blood counts were obtained with Siemens ADVIA2120i (Siemens Healthineers, Erlangen, Germany) on BD Vacutainer K2-EDTA-anticoagulated whole blood (Becton Dickinson, Franklin Lakes, NJ, US); PT was measured with Siemens Thromborel S on a Sysmex CS-5100 automated coagulometer (Sysmex Corporation, Kobe, Japan) in plasma samples obtained after centrifugation at 1500 g for 15 min of whole blood collected in 1.8 mL BD Vacutainer tubes with 3.2% sodium citrate 0,109 M; arterial blood gases were analyzed with ABL800 flex (Radiometer, Copenaghen, Denmark) on heparinized blood collected in BD syringes for arterial blood collection; procalcitonin concentrations were measured with LIAISON BRAHMS PCT II GEN on a LIAISON XL (DiaSorin, Saluggia VC, Italy) in plasma samples collected in lithium heparin BD Vacutainers. All other biochemical tests were measured with Siemens automated methods (wide-range CRP, AST, ALT, TBIL_2, ALB, CREA_2, LDPL) on ADVIA-1800 chemistry analyzers in lithium heparin plasma samples: immunoturibidmetric wide-range CRP, enzymatic methods for AST, ALT and LDH (forward reaction), colorimetric methods for creatinine (kinetic Jaffe reaction), bilirubin (vanadate oxidation method) and albumin (bromocresol green endpoint). Internal quality control and external quality assessment were implemented for all these measurands during the study period.

To diagnose SARS-CoV-2 infection, a commercial one-step reverse transcriptase-polymerase chain reaction (RT-PCR) (GeneFinder™ COVID -19 PLUS Real Real Amp Kit) was used and RT-PCR assay was performed on an Applied Biosystems 7500 Sequence Detection System (Applied Biosystems, Foster City, CA, United States).

### CT acquisition technique

CT scans were performed without contrast media injection, with one of the following scanners: 128-slice Somatom Definition Edge, Siemens Healthcare; 64-slice Ingenuity, Philips Healthcare; 16-slice GE Brightspeed, GE Medical Systems. Other details on CT acquisition technique have been previously reported [12].

### CT structured reporting and retrospective analysis

**Fig. 1:**
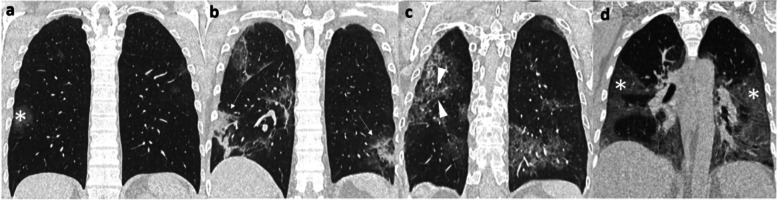
Coronal chest CT images representing the visual scoring system used to evaluate the degree of parenchymal involvement: < 20% (**a**), 20–39% (**b**), 40–59% (**c**), > = 60% (**d**). Parenchymal qualitative CT findings evaluated in this study were: ground-glass opacities (asterisks in panels **a** and **d**), consolidation (arrows in panel **b**), crazy-paving pattern (arrowheads in panel **c**)

In the period between March 13 and March 23, during routine CT reporting, each radiologist completed both the usual radiology report as well as a structured report, including the presence/absence of GGO and consolidations, and the extension of pulmonary lesions using a visual scoring system (< 20%, 20–39%, 40–59%, and ≥ 60% of parenchymal involvement) (Fig. 1) [12]. Chest CTs which were performed in this time frame were not retrospectively reviewed.

CT scans performed in the time period between February 27 and March 13 were retrospectively reviewed by an experienced radiologist. Besides the three parameters described above (GGO, consolidations, and visual scoring), data about the presence of crazy-paving pattern, pleural effusion, and mediastinal or hilar lymph node enlargement (short axis > 1 cm) were also recorded. The interrater agreement in CT disease extension evaluation has already been reported to be excellent [14].

### Statistical analyses

Laboratory results are reported as medians (interquartile range, IQR). Distribution of CT findings across multiple qualitative and quantitative (lung extension) classes are reported; the association between qualitative CT findings, clinical and demographic variables and degree of parenchymal involvement was evaluated through Fisher’s exact test. The associations between CT findings and laboratory results were evaluated with multiple linear regression models adjusted for confounders sex and age. *P* values are reported as continuous measures and no preset significance threshold was used. Analyses were performed using software packages R 3.1.0 (R Foundation for Statistical Computing, Vienna, Austria) and MedCalc 18.2.1 (MedCalc Software, Ostend, Belgium).

## Results

Included in the study were 866 patients (318 from February 27 to March 13 and 548 from March 14 to March 23). Median age was 60 years (range: 18–96); there were 527 (60.9%) males and 339 (39.1) females. Charlson comorbidity index ranged from 0 to 10, with 151 (18.4%) patients having an index greater than or equal to 1. Patients were clinically evaluated after a median of 7 days (IQR: 4–9 days) since symptom onset.

Table 1 summarizes demographic and clinical variables and CT findings, grouped according to the extension of underlying parenchymal involvement. The proportion of patients over 60 years of age increased with increasing parenchymal extension evaluated by CT imaging, going from 39.2% in individuals with extension < 20 to 62.7% in cases with extension ≥60%. The same was true for the percentage of male patients, which increased from 54.6 to 73.1% in cases with extension < 20% or ≥ 60%, respectively. At the same time, the proportion of individuals with a Charlson index greater than or equal to 1 and the proportion of deaths within 30 days of admission increased with increasing parenchymal involvement, going from 15.7 to 37.9% and from 5.0 to 43.3%, respectively. Clinical outcome was associated with the degree of parenchymal involvement evaluated by CT analysis: indeed, hospitalization rates and proportions of early (within 30 days) deaths raised from 24.8 and 5.0% in patients with parenchymal involvement < 20 to 79.1% and 43.3% in cases with ≥60% compromised lungs, respectively. Overall, 837 (96.7%) patients had GGO and 547 (63.2%) had consolidation. Retrospective review of CT revealed 126 (60.3%) cases with crazy-paving pattern; enlarged lymph nodes and pleural effusion were present in 38 (12.0%) and 27 (8.5%) patients, respectively. Each individual qualitative finding was associated with the degree of lung involvement: GGO were observed in 92.3% of patients with parenchymal extension < 20% and in 100% of patients with extension ≥60%; likewise, the proportion of patients with consolidation increased from 55.2 to 73.1% (Table 1).

**Table 1:** Summary of demographic, clinical variables and CT findings

		Parenchymal extension				
		< 20%	20–39%	40–59%	≥60%	Fisher’s exact test *p*-value
**Demographic and clinical variables**						
Age	≥60 y (*n* = 432)	133 (39.2)	175 (51.5)	82 (68.3)	42 (62.7)	< 0.0001
	< 60 y (*n* = 434)	206 (60.8)	165 (48.5)	38 (31.7)	25 (37.3)	
Sex	Male (*n* = 527)	185 (54.6)	205 (60.3)	88 (73.3)	49 (73.1)	0.0001
	Female (*n* = 339)	154 (45.4)	135 (39.7)	32 (26.7)	18 (26.9)	
Charlson comorbidity index	≥1 (*n* = 151)	50 (15.7)	48 (15.0)	28 (24.8)	25 (37.9)	< 0.0001
	0 (*n* = 667)	268 (84.3)	273 (85.0)	85 (75.2)	41 (62.1)	
Hospitalization	N (*n* = 503)	255 (75.2)	197 (57.9)	37 (30.8)	14 (20.9)	< 0.0001
	Y (*n* = 363)	84 (24.8)	143 (42.1)	83 (69.2)	53 (79.1)	
Death within 30 days	N (*n* = 773)	322 (95.0)	317 (93.2)	96 (80.0)	38 (56.7)	< 0.0001
	Y (*n* = 93)	17 (5.0)	23 (6.8)	24 (20.0)	29 (43.3)	
**CT findings**						
Ground-glass opacities	N (*n* = 29)	26 (7.7)	2 (0.6)	1 (0.8)	0 (0.0)	0.0051
	Y (*n* = 837)	313 (92.3)	338 (99.4)	119 (99.2)	67 (100)	
Consolidation	N (*n* = 319)	152 (44.8)	118 (34.7)	31 (25.8)	18 (26.9)	0.0002
	Y (*n* = 547)	187 (55.2)	222 (65.3)	89 (74.2)	49 (73.1)	
Crazy-paving pattern	N (*n* = 126)	66 (60.6)	38 (33.3)	15 (25.0)	7 (20.6)	0.0042
	Y (*n* = 191)	43 (39.4)	76 (66.7)	45 (75.0)	27 (79.4)	
Enlarged lymph nodes	N (*n* = 279)	104 (95.4)	102 (89.5)	49 (81.7)	24 (70.6)	0.0005
	Y (*n* = 38)	5 (4.6)	12 (10.5)	11 (18.3)	10 (29.4)	
Pleural effusion	N (*n* = 290)	106 (97.2)	104 (91.2)	52 (86.7)	28 (82.4)	0.0101
	Y (*n* = 27)	3 (2.8)	10 (8.8)	8 (13.3)	6 (17.6)	

Proportions of demographic/clinical variables and CT findings across CT extension classes [n (% of class column)]. Data for ground glass opacities and consolidation are obtained from the entire cohort (*n* = 866); raw numbers and frequencies for the other CT categories are calculated from patients observed until March 13 (*n* = 318)

The association between parenchymal extension and laboratory results, corrected for age and sex, is reported in Table 2. Circulating concentrations of total leukocytes, neutrophils, C-reactive protein, creatinine, AST, ALT and LDH showed a progressive increase with worsening parenchymal involvement (Fig. 1); an increase in platelets, on the other hand, was appreciable only in association with the highest burden of lung involvement. A small decrease in circulating albumin concentrations and lymphocyte counts paralleled worsening parenchymal extension, along with reduced arterial oxygen partial pressure and saturation. Finally, total bilirubin, procalcitonin and prothrombin time showed only very modest increments with the extension of lung involvement by SARS-CoV-2 infection.

**Table 2:** Association between parenchymal CT extension and laboratory data

	CT lesion extension								
	< 20% (*n* = 339)		20–40% (***n*** = 340)		40–60% (***n*** = 120)		> 60% (***n*** = 67)		
	N	Median (IQR)	N	Median (IQR)	N	Median (IQR)	N	Median (IQR)	P
**White blood cells (10^9/L)**	304	4.79 (3.84–5.91)	315	5.11 (4.02–6.50)	113	5.60 (4.33–7.85)	66	6.22 (4.66–7.97)	0.0005
**Neutrophils (10^9/L)**	265	3.02 (2.32–4.04)	291	3.64 (2.66–4.76)	101	4.13 (3.05–6.16)	62	4.79 (3.49–6.49)	< 0.0001
**Lymphocytes (10^9/L)**	264	1.14 (0.86–1.57)	290	1.05 (0.77–1.39)	100	0.89 (0.68–1.29)	62	0.85 (0.63–1.05)	0.0004
**Platelets (10^9/L)**	301	180.00 (146.51–221.25)	313	176.00 (147.29–218.75)	113	180.00 (140.19–218.25)	65	214.00 (159.00–269.25)	0.0086
**Prothrombin time (ratio)**	29	1.00 (0.96–1.05)	55	1.10 (1.02–1.15)	37	1.08 (1.02–1.19)	25	1.10 (1.05–1.14)	0.1367
**C-reactive protein (mg/L)**	299	15.00 (7.02–29.73)	315	43.02 (24.03–85.38)	112	84.60 (46.68–135.66)	64	122.50 (49.55–206.40)	< 0.0001
**Procalcitonin (μg/L)**	59	0.090 (0.05–0.12)	75	0.13 (0.09–0.20)	46	0.18 (0.11–0.41)	27	0.26 (0.11–0.60)	0.6363
**Creatinine (**μmol**/L)**	90	75.60 (67.20–91.96)	109	84.88 (67.91–95.67)	59	88.42 (74.36–114.95)	33	92.84 (79.14–128.21)	0.0162
**AST (U/L)**	68	28.50 (23.20–36.00)	90	38.50 (29.00–49.00)	58	43.00 (34.00–62.28)	29	45.00 (40.00–52.95)	< 0.0001
**ALT (U/L)**	71	25.00 (19.25–39.50)	93	30.73 (19.00–43.50)	57	32.00 (23.54–41.11)	29	30.00 (23.22–39.50)	0.0091
**Total bilirubin (μmol/L)**	63	8.55 (6.84–11.97)	86	10.26 (8.48–13.68)	51	10.26 (6.84–13.68)	30	11.12 (8.55–17.10)	0.9055
**Albumin (g/L)**	14	40.90 (38.30–42.20)	30	38.60 (36.30–41.20)	25	37.50 (35.55–39.52)	13	38.30 (32.63–40.10)	0.0077
**LDH (U/L)**	249	408.50 (358.15–487.00)	273	510.53 (422.77–599.75)	88	639.50 (474.85–783.50)	49	707.00 (496.81–896.50)	< 0.0001
**PCO2 (mmHg)**	73	35.70 (32.58–38.35)	86	34.40 (31.00–37.20)	53	33.30 (30.88–36.45)	31	36.30 (32.15–42.08)	0.6574
**PO2 (mmHg)**	73	79.50 (69.33–87.05)	85	71.00 (64.20–78.40)	53	65.50 (61.68–74.42)	31	60.80 (54.63–72.65)	0.0014
**SO2 (%)**	284	96.50 (95.00–97.80)	286	95.20 (93.80–96.50)	103	93.70 (91.80–95.78)	62	91.80 (88.30–94.40)	< 0.0001

P: p-value for coefficient of CT variable in a multiple linear regression controlled for confounders age and sex

Since a correlation was found between the extent of parenchymal involvement and the presence of individual CT findings, we were interested in evaluating whether the association between laboratory results and GGO, consolidation, crazy-paving pattern, enlarged lymph nodes and pleural effusion was independent from the degree of parenchymal involvement. Consequently, the analysis was corrected for age, sex and extension of lung lesions (Table 3). After correction, GGO were associated with reduced platelet counts and increased procalcitonin concentrations; consolidation with increased CRP and reduced oxygen saturation levels; crazy-paving pattern with reduced oxygen pressure and an increasing trend in creatinine concentrations. The presence of enlarged lymph nodes was associated with increased neutrophil counts, whereas pleural effusion with increased LDH and creatinine concentrations, reduced albumin and reduced oxygen saturation levels (Additional file 1).

**Table 3:** Association between CT findings and laboratory data (corrected for parenchymal extension)

	Ground-glass opacities			Consolidation			Crazy paving		
	N (*n* = 29)	Y (=837)	P*/P**	N (*n* = 319)	Y (*n* = 547)	P*/P**	N (*n* = 126)	Y (*n* = 191)	P*/P**
**White blood cells (10^9/L)**	5.77 (3.84–7.10) (*n* = 26)	5.10 (4.02–6.51) (*n* = 772)	0.5890/0.1833	5.00 (3.98–6.34) (*n* = 285)	5.15 (4.02–6.60) (*n* = 513)	0.1979/0.5441	5.11 (3.99–6.57) (*n* = 110)	5.29 (4.16–6.63) (*n* = 173)	0.5099/0.7682
**Neutrophils (10^9/L)**	3.52 (2.71–4.67) (*n* = 24)	3.56 (2.58–4.72) (*n* = 695)	0.7394/0.4439	3.42 (2.60–4.63) (*n* = 252)	3.61 (2.56–4.80) (*n* = 467)	0.1437/0.5918	3.57 (2.66–4.71) (*n* = 89)	4.06 (2.71–5.33) (*n* = 154)	0.4268/0.8354
**Lymphocytes (10^9/L)**	1.47 (0.81–1.93) (*n* = 24)	1.04 (0.77–1.40) (*n* = 692)	0.0887/0.3354	1.08 (0.82–1.46) (*n* = 252)	1.02 (0.75–1.39) (*n* = 464)	0.2403/0.5464	1.00 (0.72–1.31) (*n* = 89)	0.94 (0.69–1.34) (*n* = 152)	0.7478/0.2758
**Platelets (10^9/L)**	205.00 (159.50–267.75) (*n* = 25)	180.00 (145.12–222.19) (*n* = 767)	0.0422/0.0201	174.72 (149.00–221.75) (*n* = 283)	184.00 (144.00–223.61) (*n* = 509)	0.7304/0.9761	178.00 (142.75–212.25) (*n* = 109)	174.00 (139.50–222.19) (*n* = 175)	0.1109/0.3102
**Prothrombin time (ratio)**	0.95 (0.95–0.95) (*n* = 1)	1.08 (1.00–1.14) (*n* = 145)	0.3795/0.4951	1.09 (1.03–1.12) (*n* = 32)	1.07 (1.00–1.16) (*n* = 114)	0.5081/0.4801	1.04 (0.98–1.12) (*n* = 49)	1.09 (1.02–1.17) (*n* = 96)	0.8513/0.5647
**C-reactive protein (mg/L)**	7.05 (1.30–29.90) (*n* = 26)	34.45 (14.90–82.80) (*n* = 764)	0.0197/0.7841	23.80 (9.92–53.42) (*n* = 281)	43.60 (17.25–96.04) (*n* = 509)	< 0.0001/< 0.0001	37.50 (14.10–80.96) (*n* = 107)	74.70 (29.23–126.95) (*n* = 171)	0.0065/0.6653
**Procalcitonin (μg/L)**	0.09 (0.05–0.15) (*n* = 12)	0.12 (0.08–0.25) (*n* = 195)	0.0416/0.0245	0.10 (0.05–0.19) (*n* = 56)	0.13 (0.09–0.28) (*n* = 151)	0.7360/0.6694	0.11 (0.07–0.25) (n = 66)	0.14 (0.09–0.25) (*n* = 125)	0.8514/0.9498
**Creatinine (μmol/L)**	75.16 (60.39–92.40) (*n* = 13)	83.82 (69.41–98.94) (*n* = 278)	0.6436/0.8846	84.88 (65.61–95.76) (*n* = 79)	84.88 (71.53–99.03) (*n* = 212)	0.3396/0.4231	82.23 (68.08–96.82) (*n* = 104)	84.00 (71.09–102.57) (*n* = 172)	0.1572/0.0468
**AST (U/L)**	28.00 (21.50–35.25) (*n* = 11)	38.76 (28.00–50.00) (*n* = 234)	0.0709/0.5098	38.00 (27.00–49.00) (*n* = 73)	38.00 (28.40–50.00) (*n* = 172)	0.6819/0.9841	31.00 (24.00–44.25) (*n* = 85)	40.34 (30.96–52.00) (*n* = 146)	0.0061/0.0698
**ALT (U/L)**	25.00 (19.00–36.50) (*n* = 11)	29.00 (20.00–41.34) (*n* = 239)	0.2297/0.5579	29.83 (22.00–44.00) (*n* = 74)	28.00 (19.00–40.00) (*n* = 176)	0.4871/0.3426	27.00 (19.00–38.00) (*n* = 87)	29.00 (21.07–43.00) (*n* = 148)	0.0422/0.1239
**Total bilirubin (μmol/L)**	9.41 (8.55–13.68) (*n* = 10)	10.26 (7.39–13.68) (*n* = 220)	0.9687/0.8017	8.55 (6.84–11.97) (*n* = 69)	10.26 (8.55–13.77) (*n* = 161)	0.1239/0.1136	9.41 (7.46–11.97) (*n* = 81)	10.26 (8.11–13.68) (*n* = 135)	0.2978/0.2033
**Albumin (g/L)**	(*n* = 0)	38.35 (35.90–40.70) (*n* = 82)	ND	38.15 (36.75–41.45) (n = 16)	38.50 (35.70–40.40) (*n* = 66)	0.3277/0.3778	38.90 (36.20–40.55) (n = 32)	38.00 (35.55–40.55) (*n* = 49)	0.7033/0.3991
**LDH (U/L)**	376.00 (354.25–471.50) (*n* = 23)	490.00 (390.65–619.75) (*n* = 635)	0.0413/0.4449	459.00 (379.00–565.00) (*n* = 245)	502.00 (394.78–644.00) (*n* = 413)	0.0039/0.0821	469.00 (379.50–596.00) (*n* = 68)	572.00 (449.71–724.00) (*n* = 116)	0.0063/0.6203
**PCO2 (mmHg)**	40.05 (30.90–45.95) (*n* = 8)	34.60 (31.50–37.58) (*n* = 235)	0.0580/0.0781	35.80 (31.70–38.65) (*n* = 72)	34.30 (31.10–37.50) (*n* = 171)	0.6043/0.6623	34.25 (30.90–37.70) (n = 82)	34.90 (31.50–37.58) (*n* = 147)	0.7452/0.8746
**PO2 (mmHg)**	76.70 (72.35–87.15) (*n* = 8)	70.65 (63.50–80.60) (n = 234)	0.6333/0.8540	73.15 (65.60–83.70) (n = 72)	69.50 (62.40–80.00) (*n* = 170)	0.3262/0.5264	74.35 (65.30–84.80) (n = 82)	68.05 (62.20–77.30) (*n* = 146)	0.0034/0.0396
**sO2 (%)**	96.90 (94.55–98.30) (*n* = 23)	95.40 (93.50–96.80) (*n* = 712)	0.1516/0.6693	95.80 (94.03–97.10) (*n* = 275)	95.20 (93.15–96.70) (*n* = 460)	0.0005/0.0154	95.55 (93.40–96.90) (*n* = 90)	93.85 (91.70–95.70) (*n* = 150)	0.0203/0.3603

Data are reported as median (interquartile range). P: p-value for coefficient of CT variable in a multiple linear regression controlled for confounders age and sex (P*) or age, sex and CT extension (P**). *P*-values for predictor CT extension in these models were < 0.05 for total leukocytes, neutrophils, platelets, CRP, creatinine, AST, ALT, LDH, albumin and oxygen partial pressure and saturation

## Discussion

**Fig. 2:**
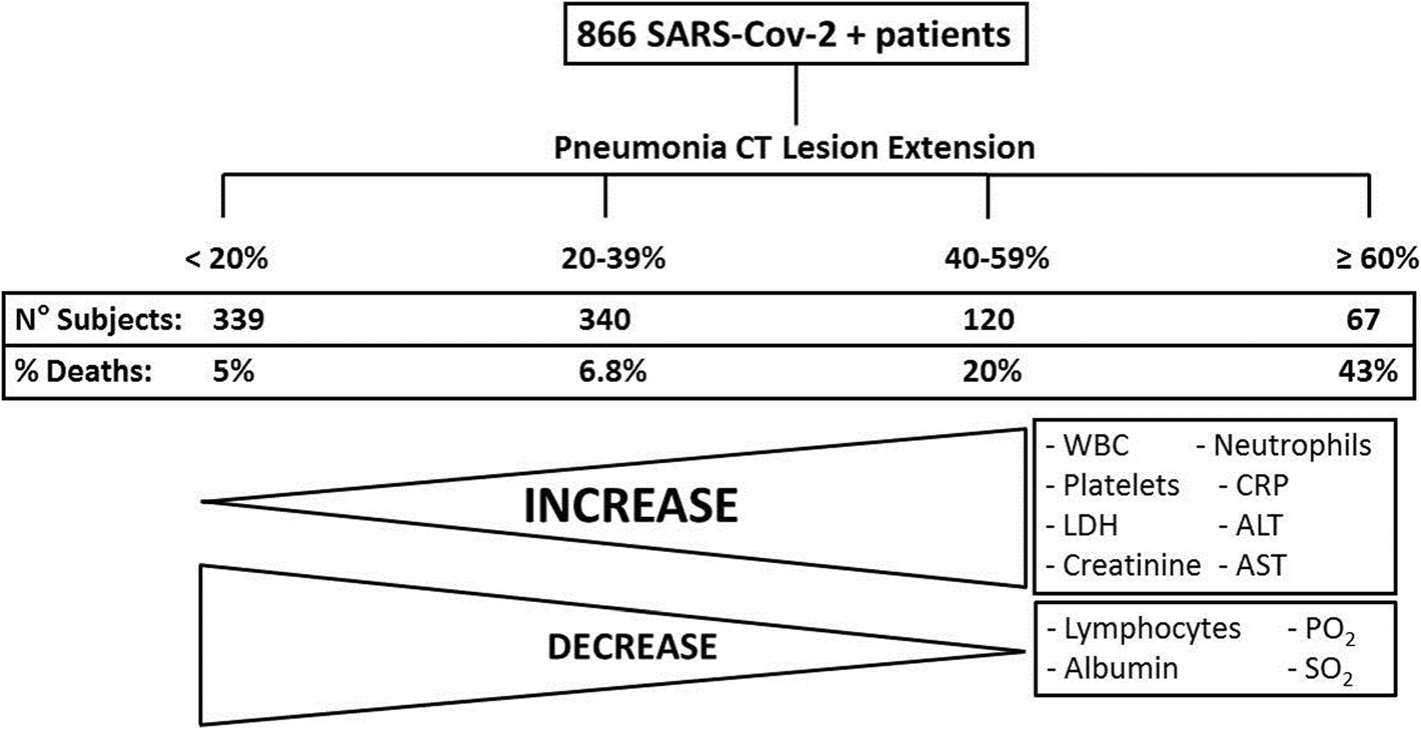
The scheme summarizes the significant variations in laboratory data related to the extension of penumonia evaluated on CT scans

We report the results of a cross-sectional study evaluating the association between CT imaging and laboratory data in a large cohort of SARS-CoV-2-infected patients observed in northern Italy during the peak of COVID-19 outbreak, taking into account quantitative and qualitative radiologic findings and their relation with changes in laboratory results reflecting inflammatory response, gas exchange and end-organ damage. The main findings of the association between laboratory data and the extension of pulmonary lesions are reported in Fig. 2. The clinical spectrum induced by SARS-CoV-2 is broad, going from subclinical infections to severe cases of pneumonia with respiratory failure, sepsis and multiple organ failure [15, 16]. In this setting, radiological and laboratory tests have established clinical roles, mainly for the prognostication of COVID-19 patients. However, the relationship between imaging findings and laboratory results is poorly studied.

First of all, we observed that the extent of lung parenchymal involvement was associated with raised inflammatory response, as suggested by increased circulating white blood cells, neutrophil counts and CRP concentrations. It has indeed been demonstrated that levels of pro-inflammatory cytokines such as IL-6 (the main stimulus for CRP production) are increased during SARS-CoV-2 infection, probably playing an immunopathologic role in cases of overproduction (so-called “cytokine storm”) [17]. However, the exact link between pulmonary lesions and inflammatory reaction remains elusive to this day. The lung itself could be the primary site of production of these pro-inflammatory mediators [18]; on the other hand, the cytokine storm could also contribute to pulmonary injury and worsening respiratory function. Like other viral diseases, the increase in procalcitonin concentrations was only marginal, probably due to the inhibitory effect of cytokines such as interferon gamma [19]. Finally, we found another aspect of dysregulated immune response associated with worsening lung involvement: a progressive reduction in circulating lymphocytes, the degree of which has been reported to be correlated with clinical severity of COVID-19 [20]. As to qualitative CT findings, consolidation was independently associated with CRP levels, confirming the findings of previous studies [21, 22], while procalcitonin levels were slightly higher in patients with GGO. Finally, we found an independent association between enlarged lymph nodes and increased neutrophil count.

The extension of parenchymal lesions was associated with impaired respiratory gas exchange and hypoxemia. This is not surprising since affected lungs show diffuse alveolar damage and interstitial edema [23]. Moreover, vascular abnormalities [24] and perfusion anomalies [25] probably concur to impair oxygen exchange. The presence of consolidation at CT, defined as an increase in parenchymal density obscuring vessels and airways, corresponds to alveolar filling by pathological fluids and cells; in COVID-19 patients this frequently presents with aspects of organizing pneumonia [26]. Hence, the independent association between consolidation and impaired gas exchange (reduced oxygen saturation level) is to be expected. Similarly, as the crazy-paving pattern consists in the superimposition of interlobular septal thickening on GGO, resulting from alveolar edema and interstitial inflammation [26], its independent relationship with reduced oxygen pressure is probably linked to alveolar-capillary interface dysfunction. Finally, compromised cardio-respiratory mechanics could further aggravate oxygen exchange in the presence of pleural effusion.

COVID-19-related coagulopathy shares similarities with diffuse intravascular coagulation, being associated with increased fibrinolytic activity (and raised D-dimer concentrations), slightly prolonged prothrombin time and reduced platelet counts [27]. We found that patients with GGO showed a modest decrease in platelet counts. GGO is the main finding in viral pneumonia and consists in a hazy increase in attenuation with preservation of bronchial and vascular structures, representing partial filling of airspaces or interstitial thickening. In our cohort, GGOs were present in almost 97% of included patients: the remaining had mostly negative CT scans, showing no prominent signs of pneumonia. In this context, the finding suggests that patients with COVID-19 pneumonia have lower platelet counts than patients without obvious pulmonary lesions. However, higher degrees of pulmonary involvement were associated with increased platelets, a finding compatible with the underlying pro-inflammatory state. At the same time, the increasing trend in prothrombin time with worsening parenchymal involvement was negligible. These findings are inconclusive and should be interpreted with caution. In particular, our data lack D-dimer concentrations, which are significantly more sensitive than platelets and PT in evaluating the presence of COVID-19 coagulopathy [28].

Finally, laboratory tests are clinically useful in recognizing end-organ damage, possibly culminating in multi-organ failure in the most severe cases. In this regard, we observed an increase in circulating levels of intracellular enzymes with worsening pulmonary involvement: in particular, LDH had the most sensitive increase, with AST and especially ALT showing only modest changes. Since these biomarkers are distributed in various tissues and organs, it is difficult to interpret their elevation and probably reflect multi-organ injury (e.g. lungs, liver, kidneys, skeletal muscle etc.). We also found a modest increase in creatinine concentrations with worsening lung involvement, while we observed only a small increasing trend in total bilirubin concentrations.

Our data are concordant with the most frequent and consistent changes previously reported in association with worsening lung involvement, concerning increased CRP and LDH concentrations and reduced lymphocyte counts and oxygen level [29]. In addition, we were able to include laboratory tests less frequently reported, such as total leukocytes, neutrophil counts and aminotransferases, which further highlighted the relationships existing between pulmonary lesions and inflammatory reaction or organ damage. While the association between CT findings and hemostatic changes has not been thoroughly investigated before, our data are inconclusive and more studies are definitely needed. Finally, unlike previous works [21], we distinguished between the associations with qualitative CT findings (e.g. GGO, consolidation etc.) and those related to the extension of the underlying lesions. In fact, since in a preliminary analysis the qualitative CT findings were found to be related with disease extension, we corrected the association between laboratory data and imaging results for the degree of parenchymal involvement, which was found to be related with the most prominent changes in laboratory results. In so doing we found interesting results that have not been previously reported, e.g. consolidation was not associated with a significant increase in circulating LDH levels but related to impaired oxygenation and increased CRP concentrations.

The main limitations of this study are that for some laboratory tests we did not have a large sample for analysis (e.g. prothrombin time, total bilirubin) and we lacked other potentially important tests such as D-dimer. This is due to the fact that we gathered real-life data regarding a novel disease during its initial outbreak, when available evidence was very scarce in guiding clinicians in appropriately requesting laboratory tests. Nonetheless, we were still able to evaluate many tests describing different possible clinical and pathophysiological derangements. Also, CT data collection was influenced by the specific period of the epidemic. Since a structured report was implemented into practice two weeks after the initial outbreak, CT scans performed in the initial time period were retrospectively reviewed. However, the reproducibility of CT visual scoring was high [8, 14], and the different data collection is unlikely to have influenced the study results. For the same reasons, we lacked information on other CT signs including findings of organizing pneumonia or interstitial lung fibrosis [30].

## Conclusions

In conclusion, pulmonary lesions induced by SARS-CoV-2 infection and evaluated by CT imaging are associated with raised inflammatory response, impaired gas exchange and end-organ damage, as evidenced by clinical laboratory data [31]. These data suggest that lung lesions probably exert a central role in COVID-19 pathogenesis and clinical presentation. More studies are needed to explore the link between lung lesions and the COVID-19 related coagulopathy.

## Supplementary Information

**Additional file 1.** Additional Table.

## Data Availability

The datasets generated and analysed during the current study are not publicly available due to privacy resctrictions, but are available from the corresponding author on reasonable request.

## Abbreviations

ALT: Alanine transaminase
AST: Aspartate transaminase
COVID-19: Coronavirus disease – 19
CRP: C-reactive protein
CT: Computed tomography
ER: Emergency room
GGO: Ground-glass opacities
IL: Interleukin
LDH: Lactate dehydrogenase
PT: Prothrombin time
RT-PCR: Reverse transcriptase-polymerase chain reaction
SARS-CoV-2: Severe Acute Respiratory Syndrome – Coronavirus – 2
