# Supplementary material for: The association between clinical laboratory data and chest CT findings explains disease severity in a large Italian cohort of COVID-19 patients
Source: BMC Infect Dis. 2021 Feb 8;21:157. doi: 10.1186/s12879-021-05855-9 (PMC7868898; doi:10.1186/s12879-021-05855-9)
Supplement: Supplementary file 1 — Additional file 1. Additional Table. [file 12879_2021_5855_MOESM1_ESM.doc]

Additional Table

|  | Enlarged lymph nodes | | | Pleural effusion | | |
| --- | --- | --- | --- | --- | --- | --- |
|  | N (n=279) | Y (n=38) | P*/P** | N (n=290) | Y (n=27) | P*/P** |
| White blood cells (10^9/L) | 5.11 (4.14-6.52) (n=247) | 5.77 (4.23-8.01) (n=36) | 0.0241/0.1154 | 5.13 (4.13-6.53) (n=256) | 5.87 (4.31-8.12) (n=27) | 0.0179/0.0491 |
| Neutrophils (10^9/L) | 3.59 (2.68-4.93) (n=211) | 5.12 (3.54-6.50) (n=32) | 0.0012/0.0191 | 3.72 (2.69- 5.17) (n=221) | 4.38 (2.83-5.76) (n=22) | 0.4072/0.4845 |
| Lymphocytes (10^9/L) | 0.98 (0.70-1.33) (n=210) | 0.87 (0.71-1.25) (n=31) | 0.9043/0.3389 | 0.97 (0.70-1.33) (n=219) | 0.86 (0.73-1.28) (n=22) | 0.0563/0.0385 |
| Platelets (10^9/L) | 177.15 (143.01-217.25) (n=247) | 171.06 (136.37-214.50) (n=37) | 0.7870/0.3971 | 175.00 (143.00-215.75) (n=257) | 161.00 (125.00-218.75) (n=27) | 0.7446/0.9816 |
| Prothrombin time (ratio) | 1.07 (1.00-1.13) (n=121) | 1.08 (1.05-1.21) (n=24) | 0.1402/0.2854 | 1.08 (1.00-1.15) (n=132) | 1.05 (1.01-1.13) (n=13) | 0.2950/0.2220 |
| C-reactive protein (mg/L) | 48.10 (20.05-107.31) (n=243) | 107.00 (44.98-152.80) (n=35) | 0.0626/0.9861 | 48.30 (20.50-107.41) (n=254) | 112.25 (57.50-153.15) (n=24) | 0.0230/0.1264 |
| Procalcitonin (µg/L) | 0.12 (0.08-0.21) (n=165) | 0.20 (0.11-0.33) (n=26) | 0.0854/0.1413 | 0.12 (0.08-0.22) (n=173) | 0.21 (0.12-0.64) (n=18) | 0.7550/0.5914 |
| Creatinine (µmol/L) | 82.23 (69.41-98.15) (n=238) | 88.95 (74.54-117.60) (n=38) | 0.0653/0.1534 | 82.23 (68.79-97.44) (n=249) | 96.38 (82.50-115.12) (n=27) | 0.0091/0.0168 |
| AST (U/L) | 39.00 (28.00-50.70) (n=200) | 35.00 (28.85-43.00) (n=31) | 0.2509/0.0363 | 38.26 (28.00-50.40) (n=208) | 36.36 (27.79-43.00) (n=23) | 0.1678/0.0686 |
| ALT (U/L) | 29.50 (20.00-43.00) (n=202) | 26.00 (19.00-32.25) (n=33) | 0.0865/0.0258 | 29.65 (21.07-42.00) (n=211) | 18.99 (15.50-32.50) (n=24) | 0.1470/0.1048 |
| Total bilirubin (µmol/L) | 10.26 (7.46-13.68) (n=184) | 9.42 (8.21-12.77) (n=32) | 0.6316/0.5953 | 10.26 (8.11-13.68) (n=195) | 8.55 (6.84-11.97) (n=21) | 0.3997/0.3755 |
| Albumin (g/L) | 38.80 (36.05-40.82) (n=69) | 36.20 (34.40-38.25) (n=12) | 0.0543/0.2649 | 38.60 (35.98-40.82) (n=73) | 36.20 (29.85-38.10) (n=8) | 0.0101/0.0161 |
| LDH (U/L) | 514.06 (394.56-653.91) (n=160) | 588.50 (458.21-773.08) (n=24) | 0.1795/0.8788 | 523.00 (397.25-689.15) (n=169) | 502.00 (414.85-530.86) (n=15) | 0.0494/0.0134 |
| PCO2 (mmHg) | 34.65 (31.45-37.50) (n=196) | 34.30 (30.48-38.90) (n=33) | 0.9309/0.9805 | 34.60 (31.38-37.73) (n=209) | 34.70 (30.00-37.15) (n=20) | 0.7325/0.7780 |
| PO2 (mmHg) | 71.00 (64.13-81.28) (n=195) | 65.70 (60.08-73.95) (n=33) | 0.1684/0.4789 | 71.00 (64.25-81.15) (n=208) | 64.30 (56.10-66.85) (n=20) | 0.0375/0.0749 |
| SO2 (%) | 94.50 (92.53-96.70) (n=207) | 93.50 (89.98-95.35) (n=33) | 0.0019/0.0376 | 94.60 (92.45-96.65) (n=220) | 92.30 (88.40-93.95) (n=20) | 0.0059/0.0121 |

Data are reported as median (interquartile range). P: p-value for coefficient of CT variable in a multiple linear regression controlled for confounders age and sex (P*) or age, sex and CT extension (P**). P-values for predictor CT extension in these models were <0.05 for total leukocytes, neutrophils, platelets, CRP, creatinine, AST, ALT, LDH, albumin and oxygen partial pressure and saturation.
